# Quasi-Elastic Neutron Scattering Studies on Hydration Water in Phospholipid Membranes

**DOI:** 10.3389/fchem.2020.00008

**Published:** 2020-01-24

**Authors:** Takeshi Yamada, Hideki Seto

**Affiliations:** ^1^Neutron Science and Technology Center, Comprehensive Research Organization for Science and Society, Tokai, Japan; ^2^Institute of Materials Structure Science/J-PARC Center, High Energy Accelerator Research Organization, Tsukuba, Japan

**Keywords:** quasi elastic neutron scattering, biological membrane, phospholipid, dynamics, hydration

## Abstract

The dynamic behavior of hydration water in phospholipid membranes has been investigated to understand the relationship between water and biological molecules using various experimental techniques. Quasi-elastic neutron scattering (QENS) is an effective method for this purpose because the dynamic behaviors of both water and lipid molecules could be identified by using selective deuteration. In addition, the measurable ranges from the 10^−12^ to 10^−9^ s time scale and the 10^−11^ to 10^−8^ m length scale are suitable to investigate the slowing down of water molecules due to their interaction with lipid membranes. In this mini-review, QENS experiments on the dynamic behavior of hydration water molecules in neighboring phospholipid membranes are summarized.

Water is essential for biological systems. For example, approximately 70% of the human body is water. This means that understanding the relationship between water and biological molecules is crucial to clarify the origins of biological functions. Many studies have been conducted on water and biological molecules such as DNA, proteins, and biological membranes (Bagchi, 2005; Berkowitz, 2019).

Many biological activities involve comprehensive transport processes through biological membranes. To understand these processes, gaining a full understanding of the structural and dynamic properties of biological membranes as well as surrounding water molecules is important. Because the composition and structure of a real biological membrane are too complex, simplified systems such as pure lipid membranes have been investigated as model systems. Because phospholipids are amphiphilic molecules, they naturally form into bilayers with hydrophobic tails inside and hydrophilic heads outside. These types of lipid membranes have been investigated using various types of experimental techniques such as nuclear magnetic resonance (NMR) (König et al., 1994; Hsieh and Wu, 1995; Nevzorov and Brown, 1997), X-ray diffraction (XRD) (Rand and Parsegain, 1989; Klose et al., 1992), neutron scattering (Pfeiffer et al., 1993; König et al., 1994; Rheinstädter et al., 2005, 2006; Seto et al., 2008; Nagao et al., 2017), dielectric spectroscopy (Antonietti et al., 1996; Klöesgen et al., 1996), differential scanning calorimetry (DSC) (Shalaev and Steponkus, 2003), dynamic light scattering (Hirn et al., 1999), and Fourier transform infrared spectroscopy (Wong and Mantsch, 1988). These experiments showed that the dynamics of membranes are hierarchical in spatial and temporal scales. It is also clear that all the dynamic behaviors strongly depend on the hydration of membranes.

To understand the relationship between phospholipid bilayers and water molecules, several experimental and computational studies have been conducted (Milhaud, 2004; Martelli et al., 2018; Calero and Franzese, 2019; Watanabe et al., 2019). Aoki and Kodama investigated the behavior of interlamellar water in phosphatidylethanolamine, phosphorylglycerol, and phosphocholine and showed the existence of both freezable and non-freezable water (Aoki and Kodama, 1998). Lazrak et al. revealed the water exchange between the inner part of the lipid membrane and the bulk region by NMR (Lazrak et al., 1987). The detailed structures of lipid membranes were shown, and the number of water molecules near the lipid headgroups were estimated through XRD (Nagle and Tristram-Nagle, 2000; Alsop et al., 2016). Molecular dynamics simulations showed that the hydration force and dynamic behavior of water molecules between lipid bilayers depend on the water structure (Marrink et al., 1993). Ultrafast polarization selective vibrational pump-probe spectroscopy of a stack of bilayers of phospholipid 1,2-dilauroyl-sn-glycero-3-phosphocholine (DLPC) showed that the dynamic behavior of water molecules between lipid membranes was different from that of hydration water at the phosphate and choline groups (Zhao et al., 2008). In another study, femtosecond mid-IR pump-probe spectroscopy verified that the structure and dynamics of water between 1,2-dimyristyl-sn-glycero-3-phosphocholine (DMPC) membranes varied with the phase transition of the DMPC bilayer from the gel phase to the liquid crystalline phase (Kundu et al., 2016). Another study investigated the dynamic behavior of water near the DMPC membranes by THz spectroscopy, and it was shown that 28 water molecules were hydrated per DMPC molecule, which was greater than the previously reported value (Hishida and Tanaka, 2011). Other THz spectroscopy experiments showed that the ratio of hydration water molecules against the total number of water molecules increased during the transition from the gel to the liquid crystalline phase (Choi et al., 2012).

Of the various types of experimental techniques, quasi-elastic neutron scattering (QENS) is a powerful method to investigate the dynamic behavior of water molecules from the 10^−12^ to 10^−9^ s time scale and from the 10^−11^ to 10^−8^ m length scale through the dynamic structure factor *S*(*Q*, ω). Because hydrogen has a huge incoherent scattering cross section throughout the atoms, neutron scattering from materials containing rich hydrogen is dominated by incoherent scattering. Although this incoherent scattering does not include the static structure, it provides information on the self-correlated dynamics of hydrogen such as the self-diffusion coefficient and rotational relaxation time (Teixeira et al., 1985; Amann-Winkel et al., 2016). The coherent and incoherent neutron scattering cross sections of deuterium are different from those of hydrogen, where the labeling of hydrogen can be performed by selective deuteration. Thus, the appropriate deuteration is useful to investigate the structural and dynamic properties of mixed compounds such as water included in materials.

Physical properties of water have attracted considerable attention for years because water shows specific features not found in other simple liquids, including a maximum density at 4°C (Zheleznyi, 1969) and divergence of heat capacity in the super-cooled region (Angell et al., 1982; Debenedetti, 2003). To elucidate the origin of the aforementioned specific features, Poole et al. showed the possible existence of a second critical point below the homogeneous nucleation temperature by molecular dynamics simulation (Poole et al., 1992). Both dynamic and structural behaviors of confined water in mesoporous materials such as mesoporous silicate have been investigated by neutron scattering because the crystallization is considered to be suppressed in the nanometer-scale space. Liu et al. reported the pressure and temperature dependence of the translational relaxation time of confined water in MCM-41 (Liu et al., 2005). Here, the translational relaxation time showed a strong-fragile transition. Yoshida et al. also reported the strong-fragile transition of confined water in MCM-41 (Yoshida et al., 2008). This transition is discussed with a hypothesis for the second critical point of water as previously described (Poole et al., 1992). The experimental results have been summarized in reviews (Mishima and Stanley, 1998; Debenedetti, 2003; Cerveny et al., 2016).

In addition, hydration water at the interface has been recognized to play a major role in functional and biological materials. Perrin et al. reported the dynamics of hydrated water in the Nafion® membrane, which is one of the most popular proton conductors, and showed the existence of two types of water characterized by local and long-range diffusion (Perrin et al., 2007). Copper rubeanate, which is a porous coordinate metal complex, exhibits high proton conductivity under high relative humidity conditions (Kitagawa et al., 2003). Yamada et al. reported that the adsorbed water in the copper rubeanate pore could be categorized into “free water,” the diffusion coefficient of which was similar to that of bulk water, and “condensed water,” the diffusion coefficient of which is 10 times slower than that of free water (Yamada et al., 2011). The free water was condensed on the pore surface with the first-order transition at 260 K, and the transition was a type of liquid-liquid transition. The dynamics of the water was slowed down due to the steric hindrance in the case of hydroxyethyl copper rubeanate (Yamada et al., 2013). Noferini et al. investigated the dynamics of hydration water in polyhydroxyethyl methacrylate gels (Noferini et al., 2019). The hydrated water was separated into immobile water associated with the polymer matrix and mobile water confined in a gel matrix. Russina et al. reported the water dynamics in a hydrophobic pore of aluminophosphate AlPO_4_-5, which provided a one-dimensional hydrophobic channel (Russina et al., 2019). The water had two diffusive motions: fast diffusion, which corresponded to the position exchange between neighboring water molecules, and slow diffusion, which corresponded to the long-range diffusion along the pore channel. These studies clearly indicated that QENS has advantages in exploring the hierarchy of the dynamics of hydration water.

As described above, the incoherent and coherent scattering cross sections are different between hydrogen and deuterium. Thus, selective deuteration enables identifying the structure and dynamics of water and phospholipid molecules separately. The first QENS results on the dynamic behavior of water molecules between lipid bilayers were published in 1994. König et al. examined oriented perdeuterated 1,2-dipalmitoyl-sn-glycero-3-phosphocholine bilayers (DPPC) at two hydration levels (*n*_*w*_ = 4−5 and 11) (König et al., 1994). Two membrane orientations (i.e., where the scattering vector was normal and parallel to the membrane) were selected to observe the anisotropy of the water dynamics in the membrane. These experiments were performed using the IRIS spectrometer at ISIS, UK, at an energy resolution of 15 μeV. The researchers showed that water molecules exhibited a slow rotational motion and no translational diffusion in the low hydration state. Jump diffusion motion similar to that of bulk water was identified in the high hydration state. The experimental results revealed homogeneous dynamic behavior, and anisotropy was not observed.

The results of the QENS experiments with selective deuteration were shown by Swenson et al. (2008). They prepared three samples: fully protonated DMPC with heavy water (DMPC/D_2_O), acyl-chain deuterated DMPC with heavy water (d54-DMPC/D_2_O), and acyl-chain deuterated DMPC with light water (d54-DMPC/H_2_O). QENS experiments were also performed with the IRIS spectrometer at a 17-μeV energy resolution. The solutions were deposited on Si(111) wafers to determine the orientation difference. The dynamic behavior of water molecules was obtained by subtracting the QENS data of d54-DMPC/D_2_O from that of d54-DMPC/H_2_O. The *S*(*Q*, ω) data were converted into the intermediate scattering function *I*(*Q, t*), and described through the Kohlrausch-Williams-Watts (KWW) stretched exponential relaxation function. From the *Q* dependence of the relaxation time, the researchers concluded that the relaxation process was that of jump diffusion, and the diffusion constant was lower than that of bulk water by only a factor of two. This was attributed to the limited energy resolution of the experiment as well as the failure to approach the sub-nanosecond time scale. No significant directional dependence was observed.

Rheinstädter et al. published the results of high-resolution QENS experiments with selective deuteration in 2008 (Rheinstädter et al., 2008). They deposited organic solution of acyl-chain deuterated DMPC (d54-DMPC) or fully protonated DMPC on Si wafers and hydrated with D_2_O or H_2_O vapor after drying. They measured four samples, d54-DMPC/D_2_O, d54-DMPC/H_2_O, DMPC/D_2_O, and DMPC/H_2_O, using the cold neutron backscattering spectrometer IN16 at the Institute Laue-Langevin, France, with an energy resolution of 0.9 μeV. The QENS data were analyzed with the aid of an all-atom MD simulation of DMPC, which showed a cooperative structural relaxation process in fluid membranes over several lipid distances.

The subsequent results of this group on the dynamic behavior of hydration water molecules were published in 2015 (Toppozini et al., 2015). The researchers prepared oriented d54-DMPC layers on Si wafers hydrated with H_2_O vapor and performed QENS experiments with the LET spectrometer at ISIS. They also analyzed the QENS data using the KWW function. They concluded that the dynamics of hydration water molecules is anisotropic and exhibit a sub-diffusive behavior in nanometer-length scales.

The most recent results were published in 2017 by Yamada et al. (2017). They prepared two samples, perdeuterated DMPC (d67-DMPC) with H_2_O and protonated DMPC with D_2_O to compare the dynamic behaviors of water and lipid molecules. The DMPC powder was mixed with the appropriate amount of water to obtain a ratio of 37 water molecules for each DMPC molecule, which corresponded to nearly the maximum amount water incorporated between lipid bilayers (Hishida and Tanaka, 2011). In contrast to the experiments previously described, the mixtures were wrapped with aluminum foil and placed in an aluminum cylinder cell. The QENS experiments were performed using the DNA spectrometer in MLF, J-PARC, Japan, at an energy resolution of 3.6 μeV. Data analysis showed that the hydration water could be categorized into three types: (1) free water with dynamic behavior is slightly different from that of bulk water; (2) loosely bound water with dynamic behavior is one-order of magnitude slower than that of free water; and (3) tightly bound water with dynamic behavior is comparable to that of DMPC molecules. The slow dynamics of loosely and tightly bound water were also reported based on molecular dynamics simulation, where the bound water forming strong hydrogen bonds to DMPC were observed to have a 20-times smaller translational diffusion coefficient than that of bulk water (Calero and Franzese, 2019). The number of the free water was 23, and the activation energy of the free water was smaller than that of bulk water. It could be related to the intermediate range order of the hydrated water reported by Martelli et al. (2018). The sum of the tightly and loosely bound water was 14, and the fraction depended on temperature. These results were quantitatively consistent with those measured by DSC (Aoki and Kodama, 1998) and by terahertz spectroscopy (Hishida and Tanaka, 2011).

QENS experiments on water and lipid membranes have the potential to relate the dynamic and structural properties of water and the functions of biological molecules. In addition, it has been known that the existence of a significant amount of loosely bound water, also referred to as “intermediate water” or “freezable water” in the literature, is considered a key factor in characterizing bio-compatible polymers (Tsuruta, 2010). The aforementioned results suggest that QENS could be a major tool used to clarify the origin of bio-related functions in biology and in material science.

## Author Contributions

All authors listed have made a substantial, direct and intellectual contribution to the work, and approved it for publication.

### Conflict of Interest

The authors declare that the research was conducted in the absence of any commercial or financial relationships that could be construed as a potential conflict of interest.
